# Effect of dietary metallo-protease and *Bacillus velezensis* CE 100 supplementations on growth performance, footpad dermatitis and manure odor in broiler chickens

**DOI:** 10.5713/ab.22.0033

**Published:** 2022-05-02

**Authors:** Cheol Ju Park, Sang Soo Sun

**Affiliations:** 1 Department of Animal Science, Chonnam National University, Gwangju 61186, Korea

**Keywords:** Animal Welfare, Broiler, Enzyme, Footpad Dermatitis, Manure Odor, Probiotics

## Abstract

**Objective:**

This study focused on the effect of dietary metallo-protease and *Bacillus velezensis* CE 100 on growth performance, carcass parameters, intestinal microflora, footpad dermatitis (FPD), and manure odor in broiler chickens.

**Methods:**

One hundred-ten (two-day-old Ross 308) broiler chicks were randomly assigned to five groups with two replicate pens. The dietary treatments were divided to control, metallo-protease groups (A1, added with 0.1%; A2, added with 0.2%) and *B. velezensis* CE 100 groups (B1, added with 0.5%; B2, added with 1.0%).

**Results:**

The feed intake was decreased in A1 and B2 compared to the other group (p<0.05). The liver weight was lower in B2 than in A2 (p<0.05). The *Salmonella* in the cecum was decreased in A2 compared to control and A1 (p<0.05). However, the lactic acid bacteria were increased in all treatments (p<0.05). The litter moisture content was decreased in A2, B1, and B2 (p<0.05). The litter quality visual score was increased in all treatments (p<0.05). The FPD score and prevalence were reduced in all treatments (p<0.05). The (CH_3_)_2_S emission was decreased in all treatments (p<0.05).

**Conclusion:**

The present study indicated that both additives improve litter quality and reduce the incidence of FPD. These findings suggest that dietary metallo-protease and *B. velezensis* CE 100 have the potential to improve the broiler chickens’ welfare.

## INTRODUCTION

Enzymes and probiotics which can improve the productivity and health of broilers are known as next-generation feed additives that replace antibiotics. Enzymes are biologically active proteins which promote the chemical breakdown of nutrients and help to increase growth rate in monogastric animals [1]. Probiotics are feed supplements composed of beneficial bacteria such as *Lactobacillus*, *Bacillus*, and *Bifidobacteria*, which can help improve poultry immunity and growth performance [2].

In the poultry industry, animal welfare issues have been raised about footpad dermatitis (FPD) and feces odor. FPD can cause necrotic lesions on the plantar surface of poultry. The main problem of FPD is an increase in the moisture content of the bedding material [3]. When the scale of dermatitis is severe, it causes pain and various behavioral disorders in the chickens, resulting in economic disadvantages [4,5]. Livestock manure contains a large amount of organic matter, it is rapidly decomposed and causes odor [6]. Odor compounds include ammonia, hydrogen sulfide, and volatile fatty acids, which harm the livestock housing environment and poultry welfare [7]. These noxious gas emissions are affected by stocking density, feed composition, litter moisture and management, etc. [6].

Dietary exogenous enzymes in broiler feed can increase body weight gain and reduce feed conversion [8]. Also, the feeding of supplemental xylanase reduced excreta ammonia and mercaptan, which had a positive effect on the broiler houses [9]. According to Gong et al [10], *B. subtilis natto*, *B. licheniformis*, and *B. cereus* were reported to improve growth performance by increasing body weight gain. *B. amyloliquefaciens* and *B. subtilis* have been reported to reduce excreta ammonia and hydrogen sulfide emission in broilers by increasing intestinal nutrient availability and inhibiting odor-causing pathogenic microorganisms [11,12]. In addition, *B. subtilis* increased the dry matter contents of chicken manure [13], and *B. amyloliquefaciens* decreased litter moisture contents and FPD under enteric pathogen challenge [14].

These previous studies suggest that enzymes and probiotics improve not only the broiler growth performance but also the FPD and manure odor emissions. On the other hand, metallo-protease is an enzyme that increases the ability to break down proteins in the presence of metal ions and can efficiently hydrolyze various protein substrates and be active at a wide range of pH and temperatures [15]. In addition, *B. velezenesis* strains have been reported that inhibit the growth of microbial pathogens and can produce bioactive metabolites such as Bacilysin and Bacillomycin-D [16], so these strains have the potential to improve the intestinal environment. These supplementations are expected to provide significant benefits to broiler productivity when used in appropriate amounts. However, the functions of metallo-protease and *B. velezenesis* strains as feed additives are not known yet, also few studies have been conducted on the effect of these single additives on poultry welfare and productivity. The objective of this study was to evaluate the impact of dietary supplemental metallo-protease or *B. velezensis* CE 100 on growth performance, intestinal microflora, litter quality, FPD, and manure odor in broiler chickens.

## MATERIALS AND METHODS

All animal experiment procedure was approved by the Institutional Animal Care and Use Committee of Chonnam National University, Republic Korea (Approval number: CNU IACUC-YB-2020-113).

### Birds and productivity

A total of 110 two-day-old Ross 308 broiler chicks were randomly assigned to five groups with two replicate pens. The dietary treatments were divided to control, metallo-protease (340,000 U/kg) group (A1, added to 0.1%/kg; A2, added to 0.2%/kg) and *B. velezensis* CE 100 (1×10^5^ CFU/mL) groups (B1, added to 0.5%/kg; B2, added to 1.0%kg). The experiment was divided into 2-phases periods (starter, d 2 to 21; finisher, d 22 to 43) and conducted for total 43 days (Table 1). Broilers were raised in pens (112×127×74 cm; 0.12 m^2^ per chicks) that flooring is covered with rice husks. According to the manual of Chonnam National University livestock breeding facility, the light was provided 24 hours until the age of 1 week, and periods of darkness were provided for 8 hours from the age of 1 week to the end of the experiment. The temperature was 38.0°C in opening day and gradually reduced to 24.0°C until the end of the experiment. The final body weight, body weight gain, feed intake, and feed conversion ratio (FCR) were measured. The weight of carcass, proventriculus, gizzard, heart, liver, small intestine, cecum, and rectum were recorded.

### Microbial analysis

Cecal contents were collected after slaughter and gradually diluted from 10^−1^ to 10^−8^ in 0.9% saline solution. Total bacteria, *E. coli*, and lactic acid bacteria were incubated in 3M petrifilim AC, EC, and LAB for 48 h at 37°C. *Salmonella* were incubated in *Salmonella-Shigella* agar (BD, Difco, Franklin Lakes, NJ, USA) for 24 h at 37°C. *Clostridium perfringens* were incubated in Tryptose sulfite cycloserine agar (MB-Cell, Seoul, Korea) for 24 h at 37°C under anaerobic conditions. Microbial colonies of each plate agar were expressed colony-forming units per gram (log10 CFU/g).

### Footpad lesion and manure odor

The severity of FPD was scored from all broilers in each pen. The dermatitis score ranged from 0 (no evidence of dermatitis) to 2 (with severe lesion) and calculated the FPD average score per pen [17]. A litter sample of 20 g from each pen (faraway feed and water line) was dried in a dry oven (60°C) for 7 days, and litter moisture contents were measured. In addition, to estimate the litter visual quality per pen, 3 independent observers scored a scale from 10 (fresh and dry) to 0 (very wet) [18].

To evaluate the manure odor, 70 g of excreta from each pen was collected and mixed 30 mL of distilled water. The samples were fermented into a 3 L suction bottle at 24°C for 2 days, and the air was injected at a constant speed (1.3 to 1.5 m/s). After fermentation period, 2 cc of gas was inhaled with a 5 mL syringe. For 4 min, NH_3_ was measured using a ODNA instrument (Nissha, Japan), and H_2_S, CH_3_SH, and (CH_3_)_2_S were measured using a ODSA instrument (Japan).

### Statistical analysis

All experimental results were processed according to the one-way analysis of variance method of the SAS (Statistics Analytical System, 9.4 Version). The significant differences among the treatment mean were analyzed with a p<0.05 statistical level using Duncan’s multiple tests.

## RESULTS AND DISCUSSION

### Growth performance

There was no difference in the final body weight, weight gain, and FCR among all treatments (Table 2). However, the feed intake was decreased in the A1 and B2 groups compared to other treatment groups except for the control (p<0.05).

Metallo-protease results were similar to previous study [19]. However, previous studies reported that supplemental exogenous enzyme (composed of xylanase, protease, amylase) and protease increased body weight gain and decreased FCR [8,20]. In poultry, enzymes can improve the growth rate and digestion efficiency by promoting the chemical breakdown of nutrients [1]. However, inconsistent with previous studies, this present study found no difference in body weight gain and FCR. Therefore, we assumed that metallo-protease did not influence broiler productivity.

*B. velezensis* CE 100 results were consistent with previous studies [21,22]. However, dietary *B. subtilis* C-3102 increased body weight gain [23]. Similarly, dietary *B. amyloliquefaciens* (20 g/kg diet) increased average daily gain, and decreased FCR [11]. Probiotics are dietary supplements composed of beneficial microorganisms, which can improve the intestinal environment and productivity of poultry [2]. In the present study, there was no difference in productivity with the supplement of dietary probiotics. These results seem to be due to feed ingredients, type of strain, and addition levels.

### Carcass characteristics

The carcass, gizzard, heart, small intestine, and rectum weights were not different in all treatments (Table 3). The liver weight was lower in the B2 group than the A2 group (p<0.05). The proventriculus weight was significantly increased in the B1 group than in the A1 and B2 groups (p<0.05).

Dietary protease increased the liver weight and decreased the heart weight [19]. However, exogenous enzyme (composed of xylanase, protease, and amylase) decreased the liver weight [8]. In this study, metallo-protease fed groups were no difference in carcass parameters. The organ weight of broilers is affected by the variety and addition level of enzyme, and the effect of metallo-protease on carcass parameters is considered insignificant.

The results of *B. velezensis* CE 100 were partially consistent with previous studies in which dietary Bacillus strains were fed [21,24]. In poultry, the liver performs various metabolic functions and is a central organ for nutrient digestion [25]. In this study, the liver weight was different between the enzyme group and the probiotics group (Table 3), but there were no significant differences with the control. Based on current results, we considered that the addition of *B. velezensis* CE 100 does not affect broiler organ weight, including the liver.

### Intestinal microflora

As shown in Table 4, the number of total bacteria, *E. coli*, and *C. perfringens* were not different in all treatments. The *Salmonella* was decreased in the A2 group compared to the control and A1 groups (p<0.05). The lactic acid bacteria increased in all treatments (p<0.05).

The gut microflora, which determines immunity and physiological status, is closely related to the productivity of broilers [26]. According to previous study with supplementary protease, there was no significant difference between *Lactobacillus*, a member of lactic acid bacteria [20]. In addition, Nian et al [27] reported that dietary xylanase increased *salmonella* in cecum. Bedford [28] suggested that exogenous enzymes can improve the digestibility of young poultry and indirectly change the intestinal microflora. In contrast, the metallo-protease in our study seems to have a positive effect on the intestinal environment by inhibiting *salmonella*, a harmful bacteria, and enhancing lactic acid bacteria.

The results of *B. velezensis* CE 100 on the intestinal microflora were partially consistent with previous studies [23,24]. However, the addition of *B. amyloliquefaciens* KB3 and *B. coagulans* produced no significant difference in the number of *Lactobacillus* and lactic acid bacteria [11,21]. Probiotics composed of *Bacillus* strains have a beneficial effect on the poultry gut, and can improve intestinal lactic acid bacteria population [24]. Moreover, *B. velezensis* strains have been reported to inhibit the growth of microbial pathogens [16]. Overall, *B. velezensis* CE 100 may create an environment where lactic acid bacteria can inhabit, and it is considered that there is no negative response on the broiler gut microbiota.

### Litter quality and footpad lesion scores

From the results in Table 5, the litter moisture contents were decreased in the A2, B1, and B2 groups (p<0.05), and the B2 group was significantly the lowest (p<0.05). The litter quality visual score increased in all treatment groups compared to the control (p<0.05). FPD prevalence and average score were decreased in all treatment groups (p<0.05).

Footpad dermatitis is a necrotic lesion that occurs on the plantar surface of poultry and negatively affects animal welfare and productivity. Occurrences of FPD are related with housing conditions, breeding management, stocking density, etc., but the leading cause is the litter moisture contents [4,5]. Previous studies reported that litter moisture contents and incidence of FPD were not significantly different with the addition of supplementary enzymes [29,30]. The low-crude protein diet significantly reduced litter moisture by 6.5% compared to the high-crude protein diet [12]. This previous research supports our findings that metallo-protease improves litter quality and FPD incidence. In addition, compared to other proteases, metallo-protease can efficiently hydrolyze a wide range of protein substrates [15]. Therefore, it is speculated that metallo-protease has the potential to enhance litter quality and decrease FPD prevalence by increasing the crude protein breakdown.

In the enteric pathogen challenge, *B. amyloliquefaciens* CECT 5940 improved litter quality and decreased the FPD severity score [14], also combination of three types *B. subtilis* and xylanase might reduce foot-pad lesion [22]. *Bacillus* spp. might help maintain the integrity of broiler gut through competitive exclusion, and it is associated with improved litter quality [14]. In addition, the excreta dry matter content increased when supplementary *B. subtilis* was fed to laying hens [13]. Collectively, *B. velezensis* CE 100 has a positive influence on the intestinal environment, which might contribute to reducing the litter moisture and severe FPD.

### Manure odor emission

As indicated in Table 6, there were no difference in the NH_3_, H_2_S, and CH_3_SH emissions among all treatments, but the (CH_3_)_2_S emission was decreased in all supplementary groups (p<0.05).

Several studies on enzymes showed that non-starch polysaccharide multi-enzyme and protease significantly reduced NH_3_ emission, but H_2_S and CH_3_SH emissions were not different [20,31]. This research was partially inconsistent with this study. On the other hand, the results for *B. velezensis* CE 100 were similar [12], who found that there was no significant difference CH_3_SH and (CH_3_)_2_S emissions when dietary probiotics composed of three *B. subtilis* strains. However, other research indicated that *B. amyloliquefaciens* KB3 and B*. subtilis* decreased NH_3_ and H_2_S emissions [11,23].

Odor compounds were decreased with an increasing number of intestinal lactic acid bacteria and gut nutrient digestibility [9,11]. In addition, litter moisture contents and type of bedding material might affect the amount of odor emissions [12]. However, all supplementary groups increased the number of cecum lactic acid bacteria (Table 4) and litter moisture contents decreased (Table 5). Therefore, it is presumed that factors other than the variety of feed supplementation, gut microbiota, and litter moisture can affect the amount of manure odor.

## CONCLUSION

In conclusion, supplementary metallo-protease and *B. velezensis* CE 100 reduced intestinal *Salmonella* or increased lactic acid bacteria, which improved the gut environment. Also, these additives reduced the moisture in the flooring, lowering the incidence of FPD. These findings imply that dietary metallo-protease and *B. velezensis* CE 100 have the potential to improve broiler chickens’ welfare.

## Figures and Tables

**Table 1:** The feed formula and chemical composition of starter and finisher period of broiler diet

Items	Starter period	Finisher period
Ingredients (%)		
Corn	48.32	55.49
Wheat grain	7.00	5.00
Wheat flour	3.00	5.00
Soybean meal	26.00	17.00
Rapeseed meal	1.00	2.00
Corn gluten	3.00	4.00
DDGS	6.50	6.50
Tallow	1.50	1.25
Limestone	1.80	1.74
Tricalcium phosphate	1.00	1.17
Salt	0.15	0.22
Methionine	0.20	0.13
Lysine	0.18	0.15
Vitamin mixture^1)^	0.20	0.20
Mineral mixture^2)^	0.15	0.15
Total	100.0	100.0
Calculate nutrients		
ME (Mcal/kg)	3.00	3.05
Crude protein (%)	20.0	18.0
Crude fat (%)	4.0	4.5
Crude fiber (%)	6.0	6.0
Crude ash (%)	8.0	8.0
Ca (%)	0.80	0.75
Available P (%)	1.50	1.50

DDGS, dry distillers grains with solubles; ME, metabolizable energy.

1) Provided vitamin mixture in broiler feed: vitamin A 18,000 mg; vitamin D 4,400 mg; vitamin E 60.0 mg; vitamin K 4.0 mg; vitamin B_1_ 0.4 mg; vitamin B_2_ 10.4 mg.

2) Provided mineral mixture in broiler feed: Fe 67.5 mg; Cu 6.75 mg; Mn 97.5 mg; Zn 90 mg; Se 0.195 mg; I 1.2 mg.

**Table 2:** Effects of dietary metallo-protease and *Bacillus velezensis* CE 100 addition for overall period on growth performance in broilers (g)

Item	Treatment^1)^				

	Control	A1	A2	B1	B2
Overall period (d 9 to 43)					
Final body weight	2,874.61±70.80	2,887.27±65.59	3,032.55±85.27	2,955.90±76.22	3,005.62±75.25
Body weight gain	2,764.17±70.06	2,781.82±64.27	2,924.00±83.69	2,847.52±74.83	2,896.48±73.56
Feed intake	4,858.31±43.55^ab^	4,780.00±23.01^b^	4,911.09±40.39^a^	4,916.37±0.56^a^	4,821.57±5.14^b^
FCR (g/g)	1.78±0.04	1.74±0.04	1.71±0.05	1.75±0.05	1.69±0.04

FCR, feed conversion ratio.

1) A1, 0.1% metallo-protease added to feed; A2, 0.2% metallo-protease added to feed; B1, 0.5% *B. velezensis* CE 100 added to feed; B2, 1.0% *B. velezensis* CE 100 added to feed.

a,b Values with different superscripts within same row are significantly different (p<0.05).

**Table 3:** Effects of dietary metallo-protease and *Bacillus velezensis* CE 100 addition on carcass and organ weight in broiler chickens (g/100 g BW)

Item	Treatment^1)^				

	Control	A1	A2	B1	B2
Carcass weight (g)	2,394.25±65.34	2,387.00±135.61	2,537.00±126.66	2,305.50±184.28	2,361.50±129.9
Proventriculus	0.46±0.07^ab^	0.35±0.04^b^	0.45±0.03^ab^	0.59±0.05^a^	0.41±0.03^b^
Gizzard	1.65±0.22	1.70±0.11	1.35±0.2	1.53±0.23	1.75±0.29
Heart	0.64±0.07	0.59±0.05	0.60±0.06	0.58±0.05	0.6±0.03
Liver	2.25±0.03^ab^	1.99±0.06^ab^	2.29±0.14^a^	1.99±0.13^ab^	1.94±0.10^b^
Small intestine	2.56±0.27	2.45±0.22	2.84±0.08	2.94±0.27	2.71±0.21
Cecum	0.43±0.07	0.47±0.05	0.47±0.07	0.52±0.07	0.44±0.03
Rectum	0.12±0.00	0.12±0.02	0.17±0.02	0.16±0.01	0.12±0.01

BW, body weight.

1) A1, 0.1% metallo-protease added to feed; A2, 0.2% metallo-protease added to feed; B1, 0.5% *B. velezensis* CE 100 added to feed; B2, 1.0% *B. velezensis* CE 100 added to feed.

a,b Values with different superscripts within same row are significantly different (p<0.05).

**Table 4:** Effects of dietary Metallo-protease and *Bacillus velezensis* CE 100 on intestinal microflora in broiler chickens (log10 cfu/g)

Item	Treatment^1)^				

	Control	A1	A2	B1	B2
Total bacteria	7.84±0.16	8.15±0.11	8.22±0.25	8.05±0.17	7.98±0.13
*E. coli*	6.87±0.18	6.89±0.13	7.27±0.29	7.00±0.20	7.07±0.23
*Salmonella*	2.60±0.13^a^	2.67±0.10^a^	1.46±0.44^b^	2.15±0.38^ab^	1.99±0.35^ab^
*C. perfrignens*	1.43±0.24	1.61±0.42	1.29±0.36	1.26±0.29	1.16±0.27
Lactic acid bacteria	7.39±0.08^c^	8.26±0.13^a^	7.80±0.08^b^	8.35±0.11^a^	7.85±0.03^b^

cfu, colony-forming unit.

1) A1, 0.1% metallo-protease added to feed; A2, 0.2% metallo-protease added to feed; B1, 0.5% *B. velezensis* CE 100 added to feed; B2, 1.0% *B. velezensis* CE 100 added to feed.

a–c Values with different superscripts within same row are significantly different (p<0.05).

**Table 5:** Effects of dietary metallo-protease and *Bacillus velezensis* CE 100 on litter moisture and footpad dermatitis in broiler chickens

Item	Treatment^1)^				

	Control	A1	A2	B1	B2
Litter moisture (%)	51.11±0.99^b^	53.72±0.57^a^	48.23±0.57^c^	39.04±1.10^d^	29.91±1.06^e^
Litter quality visual score	2.22±0.47^c^	4.28±0.54^b^	4.56±0.25^b^	7.11±0.24^a^	7.33±0.17^a^
FPD prevalence (%)	69.64±5.43^a^	24.72±7.62^b^	22.50±3.70^b^	16.94±9.02^b^	21.39±10.80^b^
FPD average score	0.93±0.18^a^	0.45±0.22^b^	0.32±0.14^b^	0.17±0.08^b^	0.22±0.10^b^

FPD, footpad dermatitis.

1) A1, 0.1% metallo-protease added to feed; A2, 0.2% metallo-protease added to feed; B1, 0.5% *B. velezensis* CE 100 added to feed; B2, 1.0% *B. velezensis* CE 100 added to feed.

a–e Values with different superscripts within same row are significantly different (p<0.05).

**Table 6:** Effects of dietary metallo-protease and *Bacillus velezensis* CE 100 on noxious gas emission in broiler chickens

Item	Treatment^1)^				

	Control	A1	A2	B1	B2
NH_3_ (ppm)	27.70±0.62	27.06±1.15	29.68±0.57	27.88±0.96	27.51±0.47
H_2_S (ppb)	0.00±0.00	0.00±0.00	0.00±0.00	0.00±0.00	0.48±0.48
CH_3_SH (ppb)	0.45±0.45	0.68±0.68	0.87±0.55	1.68±0.81	0.00±0.00
(CH_3_)_2_S (ppb)	12.63±4.82^a^	1.38±1.38^b^	0.68±0.68^b^	3.62±1.22^b^	0.00±0.00^b^

1) A1, 0.1% metallo-protease added to feed; A2, 0.2% metallo-protease added to feed; B1, 0.5% B. velezensis CE 100 added to feed; B2, 1.0% B. velezensis CE 100 added to feed.

a,b Values with different superscripts within same row are significantly different (p<0.05).
